# Bird Richness and Abundance in Urban Areas: Simulation-Based Conservation Strategies for an Italian Town

**DOI:** 10.3390/biology14010037

**Published:** 2025-01-06

**Authors:** Alessandro Ferrarini, Luca Bagni, Marco Gustin

**Affiliations:** Lipu-BirdLife Italy, Via Pasubio 3, I-43122 Parma, Italy; oasi.celestina@lipu.it (L.B.); marco.gustin@lipu.it (M.G.)

**Keywords:** bird community, bird survey, breeding birds, environmental filtering, Generalized Additive Models, quadrat sampling, sampling completeness, urban greening, urban sprawl

## Abstract

In this study, we (a) inventoried the breeding bird community of a town located in Northern Italy using quadrat sampling, (b) quantified bird richness and abundance, (c) measured sample completeness, (d) tested whether the bird community assembly was driven by environmental filtering (i.e., local properties of every single quadrat), e) explained bird richness and abundance in light of the land cover types present in each quadrat, (f) disentangled the marginal effects of every land cover type, and (g) simulated the effects on birds of different planning decisions. We recorded 36 breeding bird species, of which 17 were resident, 10 were mid-range migrants, and 9 were trans-Saharan migrators. The sampling completeness estimated ranged from 82.73% to 99.66% depending on the estimator procedure. Environmental filtering affected significantly (*p* < 0.10) the bird community assembly. Generalized Additive Models (GAMs) explained both bird richness (*R*^2^ = 91.7%) and abundance (*R*^2^ = 87.4%) satisfactorily in light of the land cover types. Simulations based on GAMs showed that local planners can largely influence bird richness in the study area, with both positive (urban greening) and negative (urban densification and sprawl) strategies.

## Simple Summary

Urbanization is one of the most significant threats to avian diversity. However, low and intermediate levels of urban development can provide birds with convenient conditions for breeding and wintering. In this study, we inventoried bird richness (number of bird species) and abundance (number of bird pairs) in an Italian town during the critical breeding period (April–June). We then investigated how bird richness and abundance were influenced by the land cover types, and simulated several changes to the land cover composition in order to predict the effects on birds. Our study demonstrates that, in the study area, environmental control on the bird community assembly is overriding, which implies that appropriate interventions on the urban land cover can bear considerable beneficial effects on the avifauna, whereas unwise planning decisions can drastically decrease bird richness and abundance. The methodological framework proposed in this study can be applied to any urban bird community.

## 1. Introduction

Multiple studies have demonstrated that higher levels of urbanization are usually associated with a lower number of breeding bird species [1,2], and that urban environments generally favor a small subset of common species while discouraging many others [3], which usually determines species turnovers along urbanization gradients [4]. It has also been suggested that the influence of urbanization on bird richness and abundance depends on the degree of modification of original habitats and the creation of new ones during urban development [5]. Lower levels of urbanization would increase bird richness by giving rise to a community characterized by the coexistence of species associated with original and new habitats, whereas higher levels would lead to a lower species richness [6,7]. Therefore, we might expect that bird richness and abundance peak at low and intermediate levels of urban development [8], as, in these propitious conditions, an urban area represents a unique type of ecosystem used by a wide range of bird species [9]. In fact, cities and towns can provide birds with a constant and predictable food supply, a shelter against extreme weather events, and suitable areas for nesting sites that play a decisive role in bird survival and reproduction [6]. The presence of green areas promotes the occurrence of urbanized woodland birds [10], whereas freshwater habitats like streams, rivers, and artificial ponds can favor waterbird species [11]. Agricultural areas in the close surroundings of cities and towns can serve as foraging and breeding habitats for open landscape bird species; in particular, ground-breeding species often associated with open habitats [12,13]. In addition, some bird species can survive in urban areas as they bear species-specific ecological traits that are advantageous for this kind of environment [14]; for instance, bird species breeding higher above the ground on buildings, or in the tree canopy or cavities, are little affected by risks like ground-dwelling predators, and disturbance by pedestrians or vagrant dogs [15,16].

Most research on urban birds has been conducted in large cities, with only few studies focused on smaller human settlements; thus, there is little knowledge about species responses to the full range of urbanization levels and whether results recorded in large cities also hold for towns [17,18]. Accordingly, in this study, we (a) inventoried the breeding bird community of a town located in Northern Italy by using fixed-area sampling squares (quadrats), (b) quantified bird richness (number of bird species) and abundance (number of breeding pairs), (c) measured sample completeness by comparing the inventory data to the predicted total richness calculated from the inventory data by using four estimator procedures of total bird richness, (d) tested whether environmental filtering (i.e., local properties of every quadrat) was the main driver of the urban bird community assembly under study, e) developed two models, fed with the data collected in the field, to explain separately bird richness and abundance in light of the land cover types present in every quadrat, (f) disentangled the marginal effects (partial response curves) of every land cover type on bird richness and abundance, and (g) utilized such models to simulate the effects on bird richness of the increase in green areas in the urban quadrats, and the road network and continuous urban fabric in the peri-urban and extra-urban ones. Our study delivers a methodological approach to assist with preserving and restoring the avian diversity in urban areas, and provides local administrations with an opportunity to test different planning scenarios and explore their implications for the conservation of urban bird communities.

## 2. Materials and Methods

### 2.1. Study Area and Bird Surveys

**Figure 1:**
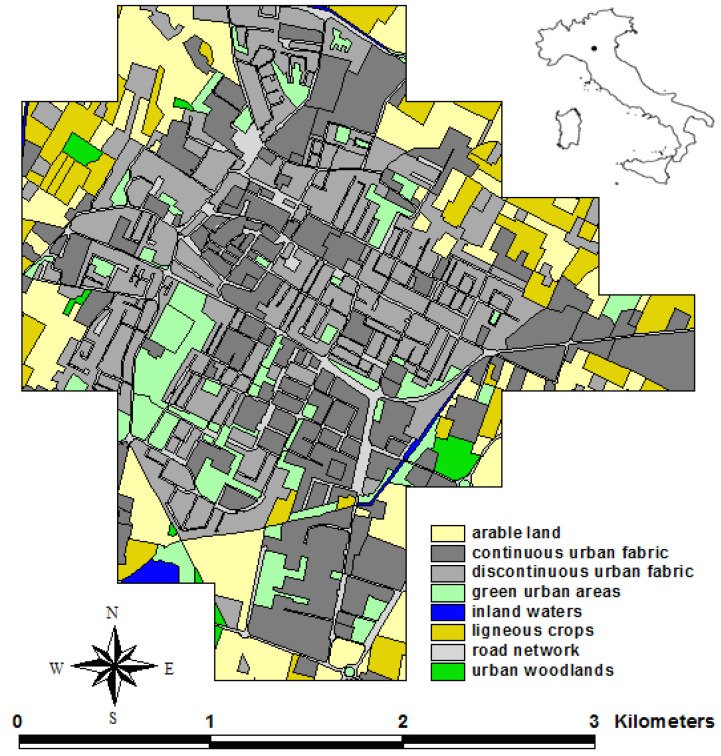
Study area (Correggio, Emilia-Romagna region, Northern Italy).

The town of Correggio is located in the lower part of the province of Reggio nell’Emilia (Northern Italy) at 33 m above sea level (Figure 1). The study area is all flat, and characterized by the prevalence of a discontinuous urban fabric (217.3 hectares; 28.98% of the study area), a continuous urban fabric (202.1 ha; 26.95%), simple irrigated arable land (116.9 ha; 15.61%), and road networks (75.8 ha; 10.09%).

We used the most popular method for sampling bird communities, i.e., fixed-area sampling squares (quadrats; [19]). The study area was divided into 30 quadrats of 500 m × 500 m each (Figure S1), including urban, peri-urban, and extra-urban (immediately adjacent to Correggio) parcels, for a total of 750 hectares (7.5 km^2^). We sampled the surrounding peripheral areas because some bird species prefer to breed in open and agricultural environments next to urban centers [20]. Each quadrat was surveyed twice, the former in April–May 2024 (sampling session 1) and the latter in May–June 2024 (sampling session 2); hence, it was possible to detect the bird species present in both the first and second halves of the breeding season. In addition, the second sampling session served to confirm and refine the first one, and detect those trans-Saharan migratory bird species that generally arrive later in the Italian breeding sites [21]. Bird surveys started after sunrise (about 5:30 A.M.) and continued until 10:00 A.M. because the singing of birds to mark territory and seek mates is more frequent in this time interval. Each observer (second and third authors) sampled half of the study area (Figure S1), going on foot. Each sampling square took between 1 and 2 h to be surveyed. On average, the observers completed 3–4 quadrats per day; thus, each sampling session required 4–5 days of bird surveys. Both observers had A3 paper maps of the sampling squares, where they marked the occurrence of the breeding males individuated through visual and sound contacts. They did not use any electronic sound recorder for the aural identification. In order to avoid uncertainties of counting the same specimen multiple times, after recording a breeding male, they observed whether the bird recorded moved from its location. Because the sampling quadrats were limited in size (500 m × 500 m), it was rather easy to observe possible movements of birds from the previously recorded location to new ones. In addition, territorial defense to warn other birds not to enter a given territory generally requires that birds to stay still, which limited the risk of counting the same specimen multiple times. The data collected in the field were then entered into a geographic information system (GIS) to be mapped and analyzed. Within the GIS, every quadrat was assigned the bird species and breeding pairs recorded, as well as the land cover classes present (available at https://geoportale.regione.emilia-romagna.it/catalogo/dati-cartografici/pianificazione-e-catasto/uso-del-suolo/layer-14, accessed on 21 October 2024).

### 2.2. Sampling Completeness

Sampling methods aim to maximize completeness, i.e., approach the actual number of species present in the study area [22]. However, given the effects of habitat complexity, identification issues, and other confounding factors, it is rarely possible to conduct an exhaustive census and quantify all bird species occurring in a given area [23,24]. Accordingly, we measured sample completeness by comparing the inventory data to the estimated total richness of the study area, calculated from the inventory data using four common estimator procedures of total bird richness: Chao1 [25], first-order jackknife [26], second-order jackknife [26], and Michaelis–Menten equation based on the Eadie–Hofstee formula [27]. We used resampling (10^4^ iterations) to prevent sample order from affecting the predictions. Finally, dividing observed richness values by predicted richness provided robust estimates of sample completeness.

### 2.3. Environmental Filtering

Once we established that sample completeness was satisfactory, we were interested in testing whether the urban bird community assembly detected was driven by environmental filtering (i.e., local properties of every quadrat). If not, any modelling effort aiming to relate bird richness and abundance to the land cover types present in the sampling squares would have been meaningless.

To test environmental filtering, inferences must be drawn from observational data [28,29]. Accordingly, we applied the methodology by [30] separately for the two sampling sessions. This test applies to presence–absence data, which consist of a list of species at quadrats (i.e., bird survey), and follows three steps: (a) compute the value of a test statistic (C-score in our study; [31]) for the matrix of observed data; (b) simulate 10^6^ presence–absence matrices equiprobably from the matrix of observed data with the observed row and column totals (i.e., randomly assembled null matrices); (c) compute the value of the test statistic (C-score) for every simulated matrix; and, (d) if the C-score value of observed data is above a chosen threshold value of the simulated distribution, then reject the null hypothesis. The null hypothesis H_0_ was that each species was equally likely to occur at all sites; i.e., quadrats were equally suitable and accessible (homogeneity of sampling squares). The alternative hypothesis H_1_ was that quadrats were non-equivalent (heterogeneity of sampling squares). If the p-value was less than 0.10, we rejected H_0_ and concluded that environmental filtering affected the co-occurrence pattern of bird species in the study area, and vice versa in the case where *p* > 0.10. We repeated Ladau and Schwager’s test three times to take into account that bird species could interact, which could partly mask the effect of environmental filtering, by assuming increasing proportions (0%, 50%, and 100%) of species interacting [30]. If H_0_ could be rejected under all these three assumptions, then we could conclude that environmental filtering was the driving force of the urban bird community under study.

### 2.4. Generalized Additive Models

Once we proved that the bird community assembly detected was driven by the local properties of every sampling square, we developed two models, fed with the data collected in the field, to explain separately bird richness and abundance in light of the land cover types present in the study area. For each quadrat, we grouped the land cover types present into eight categories (arable land, a continuous urban fabric, a discontinuous urban fabric, green urban areas, inland waters, ligneous crops, road networks, and urban woodlands) and measured their extents in hectares (*X*_1_…*X*_8_). For each quadrat, we then computed the maximum number of species (*Y*_1_) and breeding pairs (*Y*_2_) recorded in the two sampling sessions. The rationale is that we aimed to assess the amount of bird species (bird richness) and pairs (bird abundance) that could be supported by different land cover compositions, which was measurable through the maximum number of species and pairs recorded in each quadrat.

Generalized Additive Models (GAMs; [32]) are an extension of general linear models that allowed *X*_1_…*X*_8_ to be modeled as smoothing functions, which relaxed the usual assumption of linearity by permitting the form of the relationship between *X*_1_…*X*_8_ and *Y*_1_ and *Y*_2_ to take any shape as defined by the data, ranging from a straight line to curves of increasing complexity. The amount of smoothing was controlled by specifying the degrees of freedom for the smoothing function. GAMs allowed us to choose from a wide variety of distributions for *Y*_1_…*Y*_2_, and link functions for the effects of *X*_1_…*X*_8_ on *Y*_1_ and *Y*_2_. Because we were modelling count data, we constructed GAMs with a Poisson-type error distribution (appropriate for counts) for *Y*_1_ and *Y*_2_, and identity link function for *X*_1_…*X*_8_ (i.e., the linear combination of values for the predictor variables was not transformed). First, a basic model with degree of freedom value equal to 1 was developed (i.e., only linear effects of *X*_1_…*X*_8_ upon *Y*_1_ and *Y*_2_); then, we incremented the degrees of freedom for *X*_1_…*X*_8_ independently, until we reached realistic smoothing of the resulting curves and satisfactory approximation of *Y*_1_ and *Y*_2_. We applied an over-dispersion correction, which is required when the sample’s variance is greater than that of a Poisson distribution [32], in order to prevent standard errors from being underestimated and significance of model variables being overstated. We validated our models by comparing observed and predicted values separately for *Y*_1_ and *Y*_2_ in every quadrat, the *R*^2^ statistic being a measure of the proportion of the variation in *Y*_1_ and *Y*_2_ explained by the model (i.e., goodness-of-fit of predicted versus measured values). We then used the partial response curves to enable visual examination of the contribution of every land cover type to the levels of bird abundance and richness.

### 2.5. What-If Simulations

After GAMs were calibrated and validated successfully, we used results to perform several what-if simulations on bird richness by varying the proportions of land cover types. In particular, we simulated the effects on bird richness of two different, and contrasting, land cover dynamics: (a) urban expansion (i.e., the spreading of industrial and commercial units in the peri-urban and extra-urban quadrats); and (b) urban greening (i.e., an increase in green spaces in the urban quadrats).

## 3. Results

### 3.1. Bird Surveys

**Figure 2:**
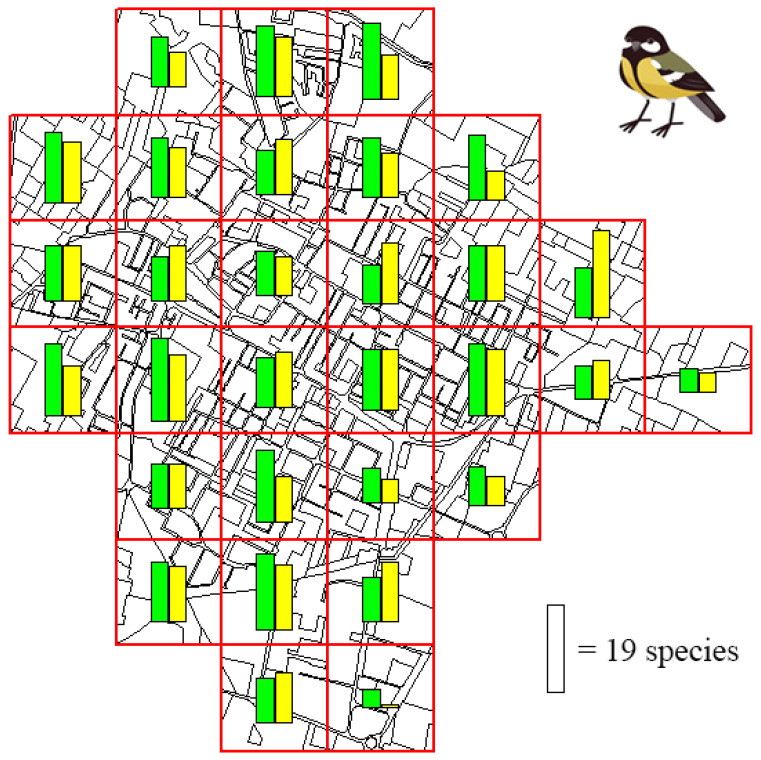
Bird richness (number of bird species) recorded in the study area during two sampling sessions (session 1: green bar; and session 2: yellow bar).

We recorded 36 breeding bird species (32 species in April–May, and 33 species in May–June) in Correggio (Table S1), mainly belonging to the Passeriformes (22 species). Of these 36 species, 17 were resident, 10 were mid-range migrants (i.e., not crossing the Sahara during the wintering phase), and 9 were long-range (i.e., trans-Saharan) migrators. We detected two SPEC 1 species (species of global conservation interest), namely, the Italian Sparrow *Passer italiae* and the Turtle Dove *Streptopelia turtur*, and five SPEC 3 species (species whose population is not concentrated in Europe, but which, in Europe, have an unfavorable conservation status), namely, the Mallard *Anas platyrhynchos*, the Common Kestrel *Falco tinnunculus*, the Eurasian Tree Sparrow *Passer montanus*, the Barn Swallow *Hirundo rustica*, and the Common Swift *Apus apus*. The highest number of bird species per quadrat was 18 in April–May, and 19 in May–June (Figure 2).

**Figure 3:**
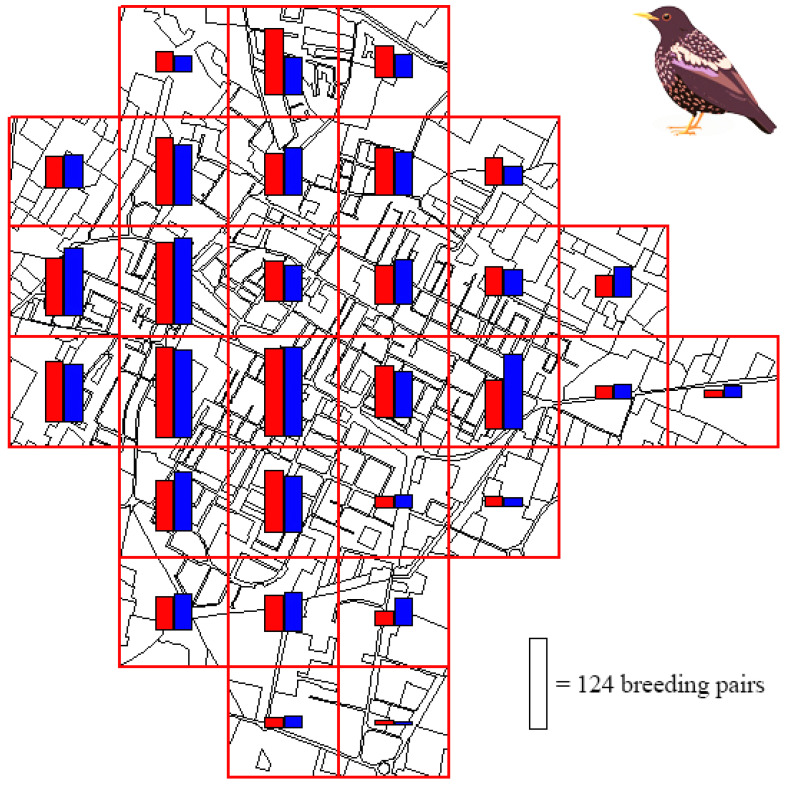
Bird abundance (number of breeding pairs) recorded in the study area during two sampling sessions (session 1: red bar; and session 2: blue bar).

The most abundant species were the Collared Dove (414 breeding pairs in April–May, and 431 in May–June), the Blackbird (342 and 352), the Wood Pigeon (115 and 192), the Starling (180 and 111), and the Great Tit (121 and 61). The locally rare species were the Common Kestrel (1 breeding pair in April–May, and 0 in May–June), the Short-toed Treecreeper (1 and 0), the Turtle Dove (0 and 1), and the Hoopoe (0 and 1). Between the two sampling dates, the main differences in the number of breeding pairs occurred for the Wood Pigeon (+77 breeding pairs in May–June compared to April–May), the Starling (−69), the Common Swift (+65), and the Great Tit (−60). The highest number of breeding pairs per quadrat was 124 in April–May, and 121 in May–June (Figure 3).

### 3.2. Sampling Completeness

The bird diversity estimated through four species richness procedures conformed well to the observed richness values (Table 1). During the first sampling session (April–May), we recorded 32 bird species, and the estimated richness ranged from 32.11 (Michaelis–Menten estimator; % completeness = 99.66%) to 38.68 (second-order jackknife estimator; % completeness = 82.73%). During the second sampling session (May–June), we recorded 33 bird species, and the estimated richness ranged from 33.68 (Michaelis–Menten estimator; % completeness = 97.98%) to 37.99 (second-order jackknife estimator; % completeness = 86.86%). Therefore, we could expect the recorded bird richness to be very close to its true value in nature.

### 3.3. Environmental Filtering

For both sampling sessions, we rejected the null hypothesis that each species was equally likely to occur at all sampling squares (Table 2). Even assuming increasing proportions (0%, 50%, and 100%) of species interacting, both the *p*-values and the upper bounds of the 95% confidence intervals for *p*-values were below the cutoff point (0.10) for rejecting *H*_0_, strengthening the conclusion of a significant result. Therefore, we accepted the alternative hypothesis *H_1_* that bird species had different probabilities of occurring at different sampling squares due to the local properties of every quadrat.

### 3.4. GAMs

**Figure 4:**
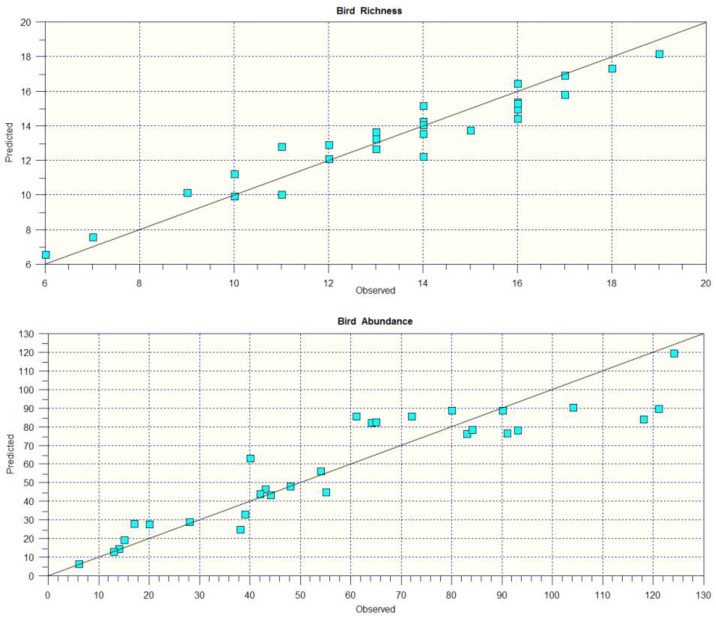
Model accuracy (observed versus predicted values in the 30 sampling squares) for bird richness (**top**) and abundance (**bottom**). The diagonal depicts the ideal situation where observed and predicted values perfectly correspond.

The model validation was successful for both bird richness (*R*^2^ = 91.7%) and abundance (*R*^2^ = 87.4%) (Figure 4).

**Figure 5:**
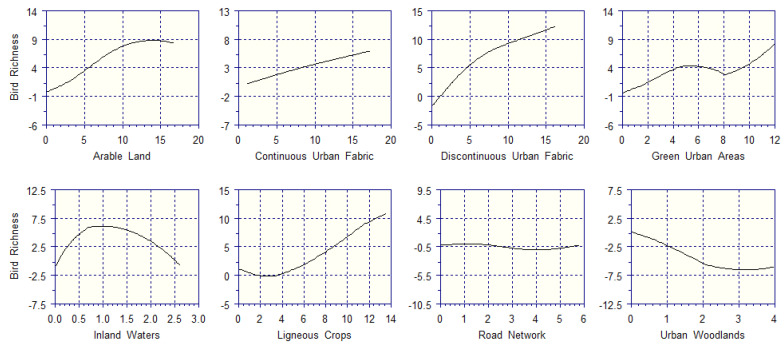
Contribution of each land cover type (*X*-axis; hectares) to the bird richness (*Y*-axis; number of bird species).

The partial response curves for bird richness (Figure 5) describe the positive and nonlinear contributions of arable land, a discontinuous urban fabric, green urban areas, and ligneous crops (vineyards). Arable land can contribute up to nine bird species to every sampling square whenever its extent reaches 15 ha (60% of quadrat size), after which its contribution is expected to stabilize, or possible decrease (because there were no sampling squares having > 15 ha of arable land, GAMs cannot solve this issue). A 15 ha size of discontinuous urban fabric adds 12 bird species to every sampling square; then, this contribution could possibly increase up to 25 hectares (100% of quadrat size); however, quadrats with > 15 ha of discontinuous urban fabric were not present; therefore, this hypothesis cannot be confirmed. Green urban areas add up to nine species to every sampling square whenever their extent reaches 12 ha (48% of quadrat size), but their contribution to bird richness remains low below 8 ha (32% of quadrat size), then increases quadratically in the 8–12 ha range. Ligneous crops provide an important support to bird richness only if they exceed 4 ha (16% of quadrat size), and can contribute up to ten bird species when they reach 14 ha (56% of quadrat size). The support of inland waters to bird richness peaks at about 1 ha (i.e., small water courses, like channels), which supports six bird species, and then declines almost linearly for larger water bodies. A continuous urban fabric provides a very low, almost linear, support to bird species, with 17 ha (68% of quadrat size) adding only six species per sampling square. Regardless of its extent, the road network decreases the number of bird species present in every quadrat. Urban woodlands bear a negative impact on bird richness, with 4 ha causing a decrease of about seven species per quadrat.

**Figure 6:**
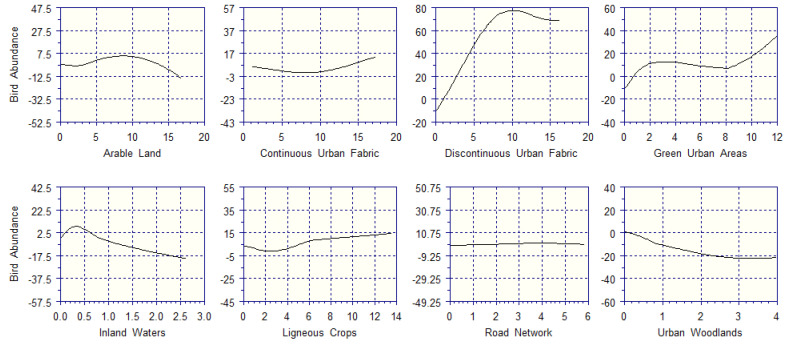
Contribution of each land cover type (*X*-axis; hectares) to the bird abundance (*Y*-axis; number of breeding pairs).

The partial response curves for bird abundance (Figure 6) depict the positive effects of a discontinuous urban fabric, green urban areas, and ligneous crops. The contribution of the discontinuous urban fabric to bird abundance peaks at 10 ha (40% of quadrat size), which adds 79 breeding pairs, then declines in the 10–15 ha range. Green urban areas add up to 38 breeding pairs to every sampling square whenever their extent reaches 12 ha (48% of quadrat size), but their contribution to bird richness remains limited (<8 pairs) below 8 ha. Ligneous crops can contribute up to 15 breeding pairs to bird abundance whenever they reach 14 ha. The contributions of arable land and inland waters to bird abundance peak at 8 ha and 0.3 ha, respectively, and then they decrease to negative values. A continuous urban fabric scarcely supports bird abundance, with 17 ha adding less than 11 breeding pairs per sampling square. Regardless of its extent, the road network decreases the number of breeding pairs in every quadrat. Urban woodlands negatively impact bird richness, with 4 ha causing a decrease of 20 breeding pairs per quadrat.

### 3.5. What-If Simulations

#### 3.5.1. Urban Expansion

We simulated four scenarios of urban sprawl in the twenty peri-urban and extra-urban quadrats: (A) 100% of continuous urban fabric (industrial and commercial units); (B) 90% of continuous urban fabric and 10% of road networks; (C) 80% of continuous urban fabric and 20% of road networks; and (D) 70% of continuous urban fabric and 30% of road networks.

**Figure 7:**
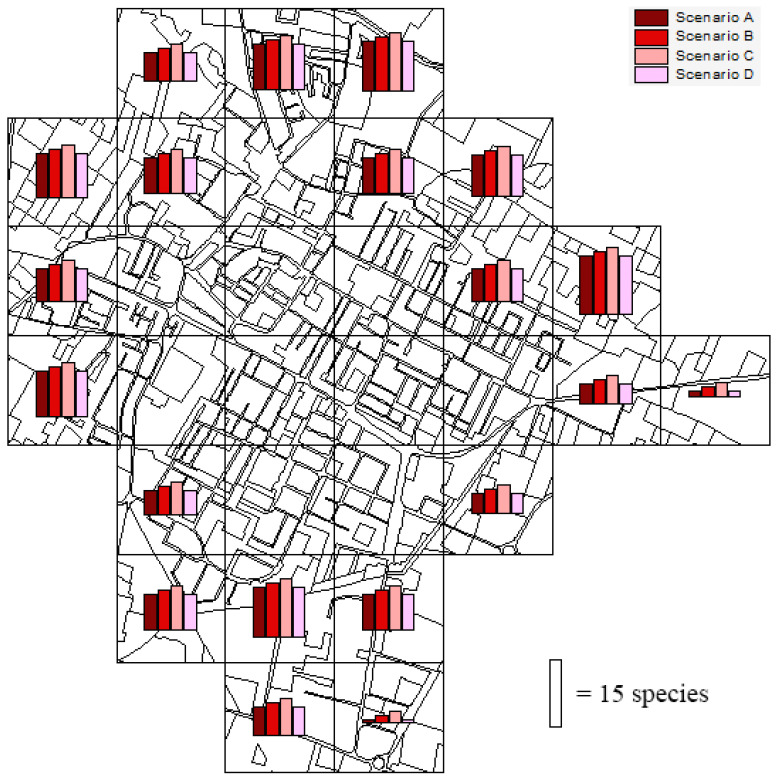
Estimated loss in the number of bird species in four different scenarios of urban expansion in the peri-urban and extra-urban sampling squares of Correggio. Scenarios: (A) 100% of continuous urban fabric; (B) 90% of continuous urban fabric and 10% of road networks; (C) 80% of continuous urban fabric and 20% of road networks; and (D) 70% of continuous urban fabric and 30% of road networks.

The estimated impacts on bird richness were quadrat-dependent (Figure 7). On average, the expected species losses per quadrat were 4.9 (±4.4 S.D.), 5.5 (±4.8 S.D.), 6.2 (±5.2 S.D.), and 4.8 (±4.4 S.D.) in the scenarios A, B, C, and D, respectively. The highest value of species loss was 15 in the scenario C for an extra-urban quadrat on the eastern side of the study area.

#### 3.5.2. Urban Greening

We simulated four scenarios of urban greening in the ten urban quadrats, i.e., increments of (E) 1 hectare, (F) 2 hectares, (G) 3 hectares, and (H) 4 hectares in urban green areas (parks, gardens, roadside trees, flowerbeds, etc.) within the continuous urban fabric.

**Figure 8:**
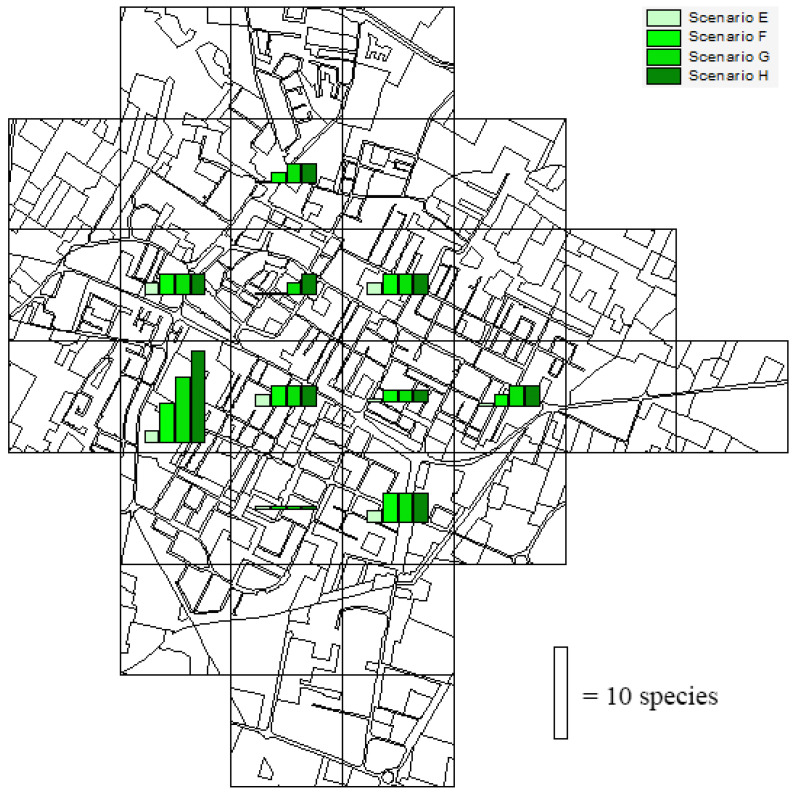
Estimated gain in the number of bird species in four different scenarios of urban greening in the urban sampling squares of Correggio. Scenarios: increments of 1 hectare (E), 2 hectares (F), 3 hectares (G), and 4 hectares (H) in urban green areas within the continuous urban fabric.

As for the what-if scenarios of urban expansion, the estimated impacts on bird richness were quadrat-dependent (Figure 8). On average, the expected gains in species richness were 0.5 (±0.4 S.D.), 1.6 (±1.2 S.D.), 2.1 (±1.8 S.D.), and 2.6 (±2.4 S.D.) in the scenarios E, F, G, and H, respectively. The highest value of species gain was 10 (scenario H) in an urban quadrat on the western side of the study area.

## 4. Discussion

The conservation of avian diversity in urban areas depends on ecologically sensitive urban planning, informed by an understanding of patterns of bird abundance and richness. Multiple studies have investigated patterns of bird richness and abundance along anthropogenic gradients such as urbanization [33] and agriculture [34]. In general, they have shown that bird diversity declines along these gradients [35,36]. However, some studies have found little influence of urbanization on bird diversity [37], with no trend in species richness, or abundance, along an urbanization gradient [38]. Other studies have even shown an increase in bird diversity in urbanized zones compared to rural and natural ones [39]. Our results suggest that these contrasting results are not surprising. In fact, we have demonstrated that, at least in the study area, both bird richness and abundance depend in a nonlinear manner on land cover types; i.e., the effect of every land cover type is different in different intervals, with some land cover types’ effects peaking at a certain value, and then decreasing. In addition, several land cover types were shown to affect differently bird richness and abundance (e.g., arable land). Because abundance measures the number of individual birds rather than species, it provides a different picture of how urban areas affect avian diversity, as compared to bird richness. This discrepancy urges the need to consider both these measures to establish a more complete picture of the processes that affect urban bird communities. The contrasting results on patterns of bird richness and abundance along anthropogenic gradients could also arise because many studies do not test sampling completeness and environmental filtering; thus, such results could be biased due to flaws in bird surveys and/or the masking effect of competition between bird species. In our study, biotic interactions did not influence the distribution of bird species in the sampling squares (at least not in a significant manner); however, the *p*-values of the test about environmental filtering were higher for the second sampling session (May–June). The urban heat island can elongate the breeding season for all bird species enabling more breeding attempts per season [6]; thus, many breeding pairs can raise two or even three broods. Consequently, the number of birds in the study area is, in all probability, higher in the second half of the breeding season due to the presence of the chicks fledged; therefore, the competition for food and space among species normally grows. This was not enough to supersede environmental filtering as the main driver of the urban bird community assembly; however, this could easily happen in other study areas. In this case, bird richness and abundance should be modeled not only in light of the land cover types present in every quadrat, but also as a function of species interactions, which normally lowers bird diversity below the levels allowed by environmental filtering [6].

### Entailments for Avian Conservation

We found that, in the study area, bird richness and abundance are significantly regulated by the land cover types, which, in turn, are good proxies for breeding and foraging opportunities, as well as threats (predation, vehicle traffic and road strikes, air pollution, and anthropogenic noise; [40,41,42]). The strong influence of the land cover composition on bird species urges us to properly plan and manage urbanized areas to support avian diversity. In the study area, bird richness is primarily favored by a discontinuous urban fabric and green areas in the urban quadrats, and by arable land and ligneous crops in the peri-urban and extra-urban ones. The discontinuous urban fabric is a complex mix of buildings, gardens, open spaces, brownfield, roadside trees, and flowerbeds; thus, it can host many bird species with different, or even contrasting, ecological requirements during the breeding period. In the study area, this land cover class hosts bird species that breed either in green areas (e.g., the Blackcap, Blue Tit, and Great Tit) or on buildings (e.g., the Common Swift, Domestic Pigeon, and Collared Dove). Green urban areas represent scattered patches of natural habitat (lawns, woody vegetation, and canopy trees) within cemented areas. Multiple studies have demonstrated that urban bird species richness increases with the size of the green space, because larger green spaces tend to have more resources and higher habitat complexity, which can generally accommodate more species [43,44]. In the study area, such positive effect increases exponentially when green urban areas exceed 8 hectares per quadrat. However, even in the case that this threshold is not reached, our simulations showed that any planning effort aimed at urban greening in the urban quadrats, besides its importance for human well-being as well as for the plant and animal species in general [45,46], is always positive for birds. The arable land in the extra-urban parcels of the study area hosts several breeding species, like, for instance, the Common Pheasant, the Swallow, and the Zitting cisticola. Interestingly, the marginal contribution of ligneous crops to bird richness was higher than the contribution of arable land. We hypothesize that the reason could be that ligneous crops are halfway between flat arable land and treed green areas, and thus provide more opportunities, in terms of higher habitat complexity, for bird species [6]. The contribution of arable land and ligneous crops to bird richness and abundance can also be indirect; i.e., bird species that breed in the urban and peri-urban quadrats can find here open areas where food (in particular, insects) is abundant and easily accessible. The presence of small (<1 ha per quadrat) water bodies (ponds) and courses (canals), including adjacent woody vegetation, promotes the occurrence of waterbird species like the Common Moorhen, whereas larger inland waters were found to be useless for bird richness. In the study area, the few large water bodies and courses are completely unvegetated (i.e., absence of hydrophytes and helophytes), which explains why they do not promote bird diversity. The continuous urban fabric supports few breeding species in the study area. High-density residential housing offers little value as a direct habitat for birds, and its presence restricts the amount of space available for more useful land-uses, such as gardens and parks, which offer a more suitable habitat [6]. In fact, our simulations evidenced that increasing the continuous urban fabric (commercial and industrial units) in the peri-urban and extra-urban quadrats can be very detrimental to bird richness. Urban woodlands and road networks disfavored bird richness. The negative effect of urban woodlands on bird richness could be surprising. However, cities and towns tend to attract synanthropic bird species with species-specific ecological traits that are advantageous for this kind of environment [6], whereas they provide scarce opportunities for bird species that depend on natural habitats (urban avoiders) because urban woodlands are limited in size and close to sources of anthropogenic disturbance (roads, and industrial and commercial units), which was exactly the case of our study area (Figure 1). In addition, the anthropogenic urban woodlands present in the study area are very dense, dark, and devoid of brushwood, and, therefore, very unlike the natural woodlands used by urban avoiders. A paved road surface is associated with various types of disturbance (e.g., traffic noise and light pollution) that are negatively correlated with bird diversity [6]; in fact, the marginal contribution of road networks to bird richness was negative regardless of its extent within quadrats.

The effects of land cover composition on bird abundance are similar as those for bird richness, except for arable land whose effect in the extra-urban parcels of the study area bears an increasing positive effect on bird richness, whereas its contribution to bird abundance becomes negative if arable land exceeds 10 hectares (40% of quadrat size). The reason is that the bird species breeding within this land cover class (e.g., Common Pheasant, Swallow, and Zitting cisticola) are scarcely abundant in the study area.

Overall, our study demonstrates that land cover composition plays a key role for this avian assemblage; thus, local planners can largely influence the avian diversity with both positive (i.e., urban greening) and negative (i.e., urban densification and sprawl) strategies. Our results evidence that bird richness and abundance in the study area can be preserved and promoted by (1) limiting as much as possible the expansion of the continuous urban fabric and road networks in the peri-urban and extra-urban quadrats; (2) avoiding the transformation of the discontinuous urban fabric into continuous one (i.e., urban densification) in the urban quadrats; (3) creating little water bodies (ponds) and courses (canals) surrounded by vegetation layers (forbs, grasses, shrubs, and trees) in every quadrat; (4) favoring the ligneous crops (vineyards and fruit trees), rather than arable land, in the peri-urban and extra-urban quadrats; (5) preferring few green urban areas larger than 8 ha, rather than many small and scattered patches, in the urban quadrats; (6) avoiding as much as possible agricultural intensification, whose negative effects on bird richness and abundance have been already demonstrated [47,48], in the peri-urban and extra-urban quadrats by favoring disposable crops (sorghum, oat, barley, and rye), alfalfa, and fallow land. The effects on birds of such strategies would be different for each quadrat, depending on the current land cover composition and planned actions, which enlightens us on the importance of a proper selection of the portions of the study area where they should be applied, which can be accomplished by using the methodological framework developed in this study.

In this study, we investigated how bird richness and abundance were shaped by the urban land cover during the critical breeding period (April–June). Further research could determine if the inclusion of winter months contributes to significant differences to such a relation. For example, the richness and abundance may show a significant drop while the migratory birds are not present in the study area, which could possibly change the way in which wintering birds rely on the land cover types.

## 5. Conclusions

Although the Italian culture is historically accustomed to considering urban areas and nature as opposite poles, towns and cities can be home to a diverse avifauna. Future quality of life can be decided precisely by the way in which we will be able to promote biodiversity in inhabited centers, by inserting harmoniously artifacts into nature and vice versa. The successful conservation of avifauna will depend on ecologically sensitive urban planning informed by an understanding of what drives the patterns of bird richness and abundance, so as to ensure that urban areas can accommodate both a growing human population and diverse bird communities. Birds are also excellent environmental indicators as they respond quickly to changes in the structure and composition of urban areas; thus, their presence and abundance also allow local planners to promptly evaluate the health status of the urban ecosystem.

In this view, the results of this study can contribute to the urban planning, design of urban green space, and wildlife management of the town of Correggio. In the face of rapid transformations in the urban environment, especially in peripheral areas subject to frequent land consumption, the administrators of this territory can now rely on a tool to reconcile human needs with those of a fundamental component of nature, i.e., avifauna. Our methodological approach (quadrat sampling, quantification of sampling completeness, test of environmental filtering, use of GAMs to explain bird richness and abundance in light of the land cover types, assessment of the marginal effects of every land cover type on bird richness and abundance, and simulation-based conservation strategies based on GAMs) is readily applicable to any urban area worldwide.

## Figures and Tables

**Table 1:** Species richness estimates. Thirty-two species were sampled during the first sampling session (April–May), and thirty-three during the second one (May–June). % completeness is the ratio of the observed bird richness to the estimated one.

Estimator	Predicted Bird Richness (Session 1)	% Completeness (Session 1)	Predicted Bird Richness (Session 2)	% Completeness (Session 2)
First-order jackknife	37.80	84.66	37.83	87.23
Second-order jackknife	38.68	82.73	37.99	86.86
Chao1	38.43	83.27	37.18	88.76
Michaelis–Menten	32.11	99.66	33.68	97.98

**Table 2:** Results about environmental filtering, separately for the two sampling sessions. The null hypothesis *H_0_* was that each species was equally likely to occur at all sampling squares, whereas the alternative hypothesis *H_1_* was that sampling squares were not non-equivalent. We repeated the test three times, by assuming increasing proportions (0%, 50%, and 100%) of species interacting.

	Sampling Session 1	Sampling Session 2
Assumption 1: 0% of species interacting		
*p*-value	*p* < 0.001	*p* = 0.037
95% confidence interval for *p*-value	0 < *p* < 0.001	0.003 < *p* < 0.074
Assumption 2: 50% of species interacting		
*p*-value	*p* < 0.001	*p* = 0.059
95% confidence interval for *p*-value	0 < *p* < 0.001	0.031 < *p* < 0.084
Assumption 3: 100% of species interacting		
*p*-value	*p* < 0.001	*p* = 0.064
95% confidence interval for *p*-value	0 < *p* < 0.001	0.040 < *p* < 0.094

## Data Availability

The data are available from the first author upon reasonable request.

## Supplementary Materials

The following supporting information can be downloaded at: https://www.mdpi.com/article/10.3390/biology14010037/s1, Figure S1: Google Earth image of the study area with the sampling squares overlapped and labelled; Table S1: Bird richness and abundance recorded in the sampling squares.
