# Supplementary material for: Bird Richness and Abundance in Urban Areas: Simulation-Based Conservation Strategies for an Italian Town
Source: Biology (Basel). 2025 Jan 6;14(1):37. doi: 10.3390/biology14010037 (PMC11762845; doi:10.3390/biology14010037)
Supplement: Supplementary file 1 [file biology-14-00037-s001.zip › biology-3387745-supplementary.pdf]

Supplementary materials

Article

Bird Richness and Abundance in Urban Areas: Simulation-Based Conservation Strategies for an Italian Town

Alessandro Ferrarini *, Luca Bagni and Marco Gustin

Lipu-BirdLife Italy, Via Pasubio 3, I-43122 Parma, Italy; oasi.celestina@lipu.it (L.B.); marco.gustin@lipu.it (M.G.)
* Correspondence: sgtpm@libero.it; Tel.: +39-0521-1910728

Figure S1. GoogleEarth image of the study area with the sampling squares overlapped and labelled. Yellow and green colors depict the quadrats sampled by each observer (second and third authors).

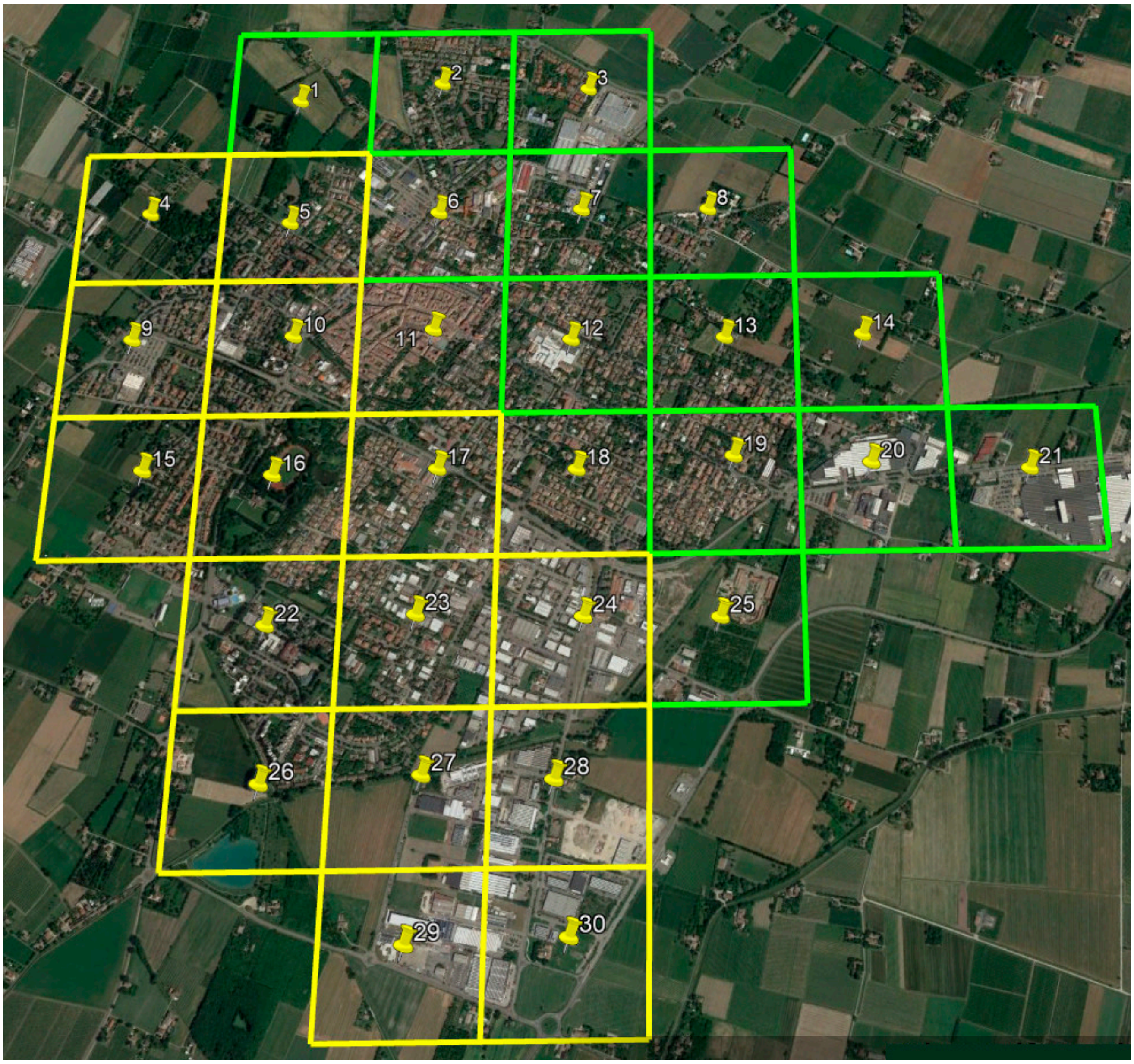

Table S1. Bird richness and abundance recorded in the sampling squares, separately for each sampling session. IDs refer to the labels in Figure S1.

Sampling squares	Sampling session 1		Sampling session 2	
ID	Bird richness	Bird abundance	Bird richness	Bird abundance
1	12	28	9	21
2	16	90	14	50
3	17	43	11	31
4	16	43	14	44
5	14	91	12	81
6	11	56	13	64
7	14	65	11	59
8	15	39	8	26
9	13	78	13	93
10	11	113	13	118
11	11	55	10	49
12	10	53	14	61
13	13	40	13	36
14	12	29	19	42
15	16	83	12	79
16	18	124	15	119
17	12	119	13	121
18	14	72	14	63
19	16	68	15	104
20	9	18	10	20
21	7	8	6	14
22	11	69	11	80
23	16	84	11	76
24	9	16	7	17
25	10	13	8	12
26	14	45	13	48
27	17	49	15	54
28	11	20	14	38
29	11	14	12	15
30	6	6	3	3
